# Stigma and Inequity in Tuberculosis Transmission and Control in the Philippines

**DOI:** 10.3390/pathogens14121226

**Published:** 2025-11-30

**Authors:** Gene Khyle Francis Uy Galvez, Jasmine Soco Interior

**Affiliations:** Mapúa School of Medicine, Mapúa University, Makati City 1002, Metro Manila, Philippines; jasmine.s.interior@gmail.com

**Keywords:** tuberculosis, stigma, social determinants of health, institutional discrimination

## Abstract

Tuberculosis remains endemic in the Philippines despite decades of biomedical progress under the WHO End TB Strategy. This persistence reflects not a failure of medicine, but of systems that treat tuberculosis as a biomedical issue rather than a social one. While public health programs recognize community factors, stigma is still framed as a problem of awareness rather than a structural outcome of health institutions. Practices of isolation, surveillance, and labeling have normalized fear and exclusion, shaping how communities perceive and respond to the disease. By pathologizing patients rather than confronting inequities, institutions perpetuate the very stigma that hinders diagnosis and treatment. To end tuberculosis, national frameworks, especially in low- and middle-income settings, must become stigma-responsive by embedding social trust, accountability, and equity as measurable goals alongside cure rates. Only then can the End TB Strategy’s promise of universality and dignity be realized.

## 1. Introduction

Despite decades of biomedical advances, tuberculosis (TB) remains a significant public-health challenge in the Philippines. In 2023, the Philippines reported an estimated 739,000 people with tuberculosis, corresponding to an incidence of approximately 643 cases per 100,000 population and a mortality rate of roughly 32 deaths per 100,000, placing the Philippines among the countries with the highest global TB burdens. TB treatment coverage was estimated at 78%, indicating ongoing gaps in early detection and access to care. Moreover, the country also has one of the highest burdens of MDR/RR-TB globally, with the incidence estimated at 25 per 100,000, underscoring the growing challenge of drug resistance [1].

## 2. Screening, Surveillance, and the Limits of Biomedical Progress

The Philippines implements a systematic screening strategy as part of the National Tuberculosis Control Program, where screening is conducted both in health facilities through intensified case finding in communities and workplaces through active case finding, especially in high-risk settings. In health facilities, adults are screened for cardinal signs and symptoms such as cough, unexplained fever, weight loss, and night sweats. Moreover, individuals are recommended to undergo annual chest X-ray screening. Under the National TB Program, suspected cases are ideally confirmed using Xpert MTB/RIF, which detects both *Mycobacterium tuberculosis* and rifampin resistance. However, implementation varies across settings due to resource limitations, with many local health centers relying on symptom screening and radiography as initial tools. 

On the other hand, community-based screening prioritizes urban poor communities, construction and transport workers, as well as individuals in detention facilities. Moreover, annual screening is ideally conducted among healthcare workers, with contact tracing ideally carried out for household contacts [2]. However, routine implementation remains inconsistent due to variable local capacity and priorities.

In 2025, the Department of Health reported a decrease in chest X-ray screenings by 56%, with new case notifications dropping to 6% [3]. These figures reveal not only operational disruptions but also the deeper reality that technological and diagnostic innovations do not automatically translate into better healthcare-seeking behaviors or treatment adherence. TB control continues to falter because stigma, which is often dismissed as a social or cultural problem, is in fact institutionalized within health systems. By treating stigma as peripheral rather than measurable, policy frameworks reproduce inequities rather than addressing them.

The WHO End TB Strategy situates TB elimination within a biomedical paradigm that prioritizes detection, treatment success rates, and pharmacological innovation [4]. While Lau et al. (2020) describe how continuity of community presence can influence participation in active case-finding, their study did not directly assess stigma or trust in health workers [5]. Evidence from qualitative work in the Philippines shows that decisions to seek diagnosis and treatment are shaped by concerns about blame, loss of privacy, and disruptions to work and home responsibilities, which can discourage screening and follow-through [6,7]. In this context, reductions in screening cannot be explained solely by epidemiology, but also by uneven community engagement and variable experiences of respect and safety during healthcare encounters [6,8]. Programs that foreground biomedical progress without simultaneously strengthening social trust and accountability risk producing the appearance of success while excluding those most vulnerable to infection.

## 3. Social Determinants and Structural Vulnerabilities

Tuberculosis is widely known as a disease of poverty, but that phrase conceals its structural roots. Overcrowded housing, undernutrition, and precarious employment remain persistent determinants [9]. In the Philippines, 32.0% of families in rural areas lived in housing with a floor area of less than 30 square meters, compared with 29.0% in urban areas, according to the 2022 Annual Poverty Indicators Survey [10]. Nutrition and food security may also be factors, according to the 2021 Expanded National Nutrition Survey, which found that 33.4% of households were moderately to severely food insecure, with 7 out of 10 Filipinos concerned about not having enough food [11]. Working and living conditions also shape vulnerability to tuberculosis in the Philippines. According to the Employment Demand and Statistics Division of the Philippine Statistics Authority, approximately 18.5% of the employed population in 2016 were engaged in precarious work, characterized by casual or project-based employment in which workers lack job security and income stability [12].

## 4. Social Determinants and Structural Vulnerabilities

Institutional practices may unintentionally attach moral meaning to clinical classifications, where patients with active pulmonary TB are not only identified as “infectious” in a biomedical sense but are perceived as risky or undisciplined. Such judgements that frame patients as morally responsible for their illness can reinforce social distancing, blame, or exclusion in workplaces and clinical settings [7]. Paterson et al. (2025) emphasize that stigma is not merely attitudinal; it is reproduced and sustained by systems that categorize and manage patients, as well as how these are reinforced within communities [13]. In the Philippines, the decentralization of health services has led to significant gaps between local government units (LGUs), resulting in inequities in tuberculosis program delivery. Moreover, referral pathways are often fragmented, and limitations in insurance coverage, such as PhilHealth, pose additional barriers to diagnosis and treatment continuity [8]. In this light, inequity does not merely contribute to the tuberculosis endemic; it is an outcome of the fragmented and reactive system that sustains it.

## 5. Lived Realities and Community Responses to TB Stigma

Recent work in the Philippines highlights how stigma is reinforced not only through fear of infection but also through expectations of moral responsibility and patient compliance. Lacsa (2025), for example, underscored how stigma is intensified in prison environments, where a culture of surveillance, isolation, and regimented treatment persists despite overcrowded conditions and limited healthcare that, ironically, worsen disease transmission in the first place [6]. As a result, this normalizes tuberculosis as a disease of containment rather than care. Moreover, Zimmerman et al. (2022) demonstrate that care-seeking decisions among Filipinos are shaped by trade-offs between accessing treatment and preserving the status quo, which involves maintaining social belonging, privacy, and economic stability [7]. The labels “infectious” or “irresponsible have highly disincentivized many from disclosing their disease or seeking their treatment. In these contexts, contracting tuberculosis is experienced and expressed less as a biomedical condition but more as an outcome of their fate, which further naturalizes stigma.

Toheeb et al. (2024) document how communities in Southern Mindanao acknowledged stigma as a sociocultural reality rather than normalizing it [14]. Faith-based institutions reframed tuberculosis as a shared community responsibility through dialogue and counseling, positioning them as mediators of trust. Yet these efforts remain constrained by persistent cultural and institutional barriers that limit their reach. This highlights that while individual behavior change is necessary, it cannot substitute for systemic reform. Without structural support, even the most trusted community initiatives struggle to sustain impact. Within Philippine health systems, the absence of confidentiality safeguards, social support mechanisms, and continuity of care perpetuates the very conditions that drive silence and avoidance. For health practitioners, acknowledging stigma as a consequence of institutional failure rather than a lack of awareness is the first step in reorienting tuberculosis management around the principles of trust, accountability, and equity.

## 6. Regional Perspectives on Stigma and Program Design

In other high-burden settings, where comparable patterns are observed, stigma is not merely a cultural phenomenon but is instead shaped by program design and institutional environments. In Indonesia, Fuady et al. (2024) explored the association of stigma with depression, reduced quality of life, and diminished social participation, highlighting the need for integrated psychosocial support and recovery within TB programs [15]. Similarly, in Nepal, Dixit et al. (2024) observed that patients diagnosed through active case-finding strategies experienced higher levels of both enacted and internalized stigma than those diagnosed passively [16]. With this, it is also imperative to consider the role of the detection method in shaping how disease identity is socially perceived. These parallels illustrate that tuberculosis stigma is linked not just to existing social and cultural factors, but also to how TB control strategies are implemented, making stigma-responsive policy design essential to improving health outcomes.

## 7. Lessons from the HIV Response and Policy Implications

Given current paradigms, the national tuberculosis program should measure the success of interventions not only through treatment completion or diagnosis rates, but also through reductions in social exclusion. In contrast, the HIV Prevention and Control Operational Plan 2018–2020 integrated stigma monitoring and community-led accountability through behavioral surveillance and stigma-reduction programs under the National AIDS and STI Prevention and Control Program (NASPCP). This approach, supported by multisectoral representation from agencies such as the National Youth Commission, the Department of Education, the Department of Social Welfare and Development, and the Department of Labor and Employment, frames HIV not merely as a disease but as a social issue shaped by contexts and communities [17]. Consequently, HIV testing and diagnosis coverage have rebounded to pre-pandemic levels, reaching 66% among estimated people living with HIV [18]. However, HIV activism emerged in a context of strong civil society mobilization, sustained global financing, and legal protections for patient rights. An equivalent level of political mobilization or community funding has not yet supported the TB response in the Philippines. Thus, while HIV programs offer valuable models for stigma reduction and community ownership, translating these approaches to TB requires parallel investments into community leadership, social protection, and long-term program infrastructure rather than direct replication.

## 8. Toward Stigma-Responsive Tuberculosis Policy

Measurement frameworks for TB stigma already exist, including the USAID/Challenge TB Stigma Measurement Guidance (2021–2023), which provides indicators for assessing stigma at the individual, community, and health system levels. However, such tools remain underutilized in the Philippine TB program, limiting systematic monitoring and response. Yet, TB control remains trapped in a biomedical framework that measures cure but not dignity. The path forward lies in a stigma-responsive approach towards tuberculosis. Stigma must be recognized a measurable policy variable that can be addressed through targeted interventions such as embedding stigma assessment tools in national TB surveillance as well as performance indicators, empowering community health workers to deliver stigma-sensitive education, implementing social and behavioral change communication (SBCC) campaigns through multidisciplinary and intersectoral approaches, and embedding stigma-reduction budgets within tuberculosis funding lines and eventually PhilHealth, the country’s national health insurance program. Through these reforms, equity would subsequently become a measurable objective rather than an abstract aspiration.

## 9. Conclusions: Embedding Trust and Equity in TB Elimination 

Reducing tuberculosis cannot rely solely on detection and treatment outcomes; it also requires addressing the social and relational environments in which care is sought. Stigma operates across multiple levels, including interpersonal, community, workplace, and institutional, with each level requiring a tailored approach. Integrating routine stigma monitoring, enhancing confidentiality and patient protection, investigating community-engaged communication, and strengthening social support mechanisms can help ensure that accessing care does not threaten livelihood, dignity, or belonging. Embedding trust and equity into program design is not only consistent with the goals of the End TB Strategy. Still, it is essential for ensuring that diagnosis and treatment are genuinely accessible to all.

## Abbreviations

The following abbreviations are used in this manuscript:

TB	Tuberculosis
MDR/RR-TB	Multidrug-resistant or rifampicin-resistant TB
MTBC	*Mycobacterium tuberculosis* complex
RIF	Rifampin
WHO	World Health Organization
HIV	Human Immunodeficiency Virus
PhilHealth	Philippine Health Insurance Corporation
